# Influencing factors for the increased inter-arm systolic blood pressure difference among populations of different genders

**DOI:** 10.3389/fcvm.2026.1767694

**Published:** 2026-03-04

**Authors:** Jing Cheng, Bao Zhang

**Affiliations:** Department of Endocrinology (Clinical Nutrition), The First Affiliated Hospital of Anhui Medical University, Hefei, Anhui, China

**Keywords:** ankle-brachial index, diastolic blood pressure, gender, inter-arm systolic blood pressure difference, neck circumference

## Abstract

**Objective:**

To explore the influencing factors for the enlargement of inter-arm systolic blood pressure difference (IASBPD) between different genders.

**Methods:**

A retrospective analysis was performed on the data of individuals who underwent body composition testing in our department and completed limb arterial blood pressure measurement, biochemical examinations, and other relevant tests at the physical examination center or outpatient clinic of our hospital between September 2019 and December 2022. The participants were divided into two groups based on whether the inter-arm systolic blood pressure difference (IASBPD) was increased: the IASBPD≥10mmHg group and the IASBPD < 10mmHg group. The influencing factors for an increased IASBPD were analyzed in the overall population as well as in different gender subgroups.

**Results:**

Participants with IASBPD≥10mmHg exhibited higher obesity-related indicators, including body mass index (BMI), waist circumference (WC), neck circumference (NC), abdominal circumference (AC), and visceral fat area (VFA) (*P* < 0.05). Additionally,the group with an IASBPD≥10mmHg had a higher proportion of males, elevated levels of white blood cells, monocytes, and lymphocytes, as well as a higher prevalence of hypertension and dyslipideia (*P* < 0.05). IASBPD was positively correlated with NC, age, and lymphocytes, and negatively correlated with Ankle-Bronchial Index (ABI). An increased ABI was a protective factor for IASBPD in the general population, while an increased NC and hypertension were risk factors for an increased IASBPD. However, an increase in NC is a risk factor for an elevated IASBPD in males, while advanced age is a risk factor for increased IASBPD in females.There are differences in IASBPD values between males and females in the quartile groups stratified by NC and age. ABI-left, right and left upper arm systolic blood pressure all exhibit certain predictive performance for the increase in IASBPD in both male and female populations. The cut-off values of NC and age for predicting the elevation of IASBPD were 37.75 (sensitivity: 66.1%, specificity: 53.5%) and 49.5 (sensitivity: 77.0%, specificity: 47.5%), respectively.

**Conclusion:**

The incidence of increased IASBPD is higher in the male population. An increase in NC and advancing age are identified as risk factors for IASBPD elevation in the male and female populations, respectively.

## Introduction

Inter-arm blood pressure difference (IAD) refers to the phenomenon of unequal blood pressure in both upper limbs, which can occur in both normal and hypertensive individuals. IAD includes IASBPD and inter-arm diastolic blood pressure difference (IADBPD). IASBPD≥10mmHg is an indicator of unhealthy cardiovascular disease, a difference of 10mmHg is considered the upper limit of the normal value for IASBPD (1). Subjects with a significant abnormal IAD were more likely to report obesity and were significantly had a greater body mass index (BMI) (2), neck circumference (NC) (3), visceral fat accumulation (VFA) (4). IAD is closely related to arterial stiffness and systolic blood pressure, which can provide a more comprehensive assessment of cardiovascular risk. Carotid-femoral pulse wave velocity (CF-PWV) is considered the gold-standard measurement of arterial stiffness, patients with IASBPD≥10mmHg exhibited significantly higher CF-PWV, the partial association between IASBPD and cardiovascular risk may be mediated by arterial stiffness (5). Findings have indicated an association between IASBPD and diabetic retinopathy (DR) as well as proteinuria in diabetic patients, and IASBPD may be regarded as a surrogate marker for vascular complications in patients with type 2 diabetes mellitus (6). People with IAD≥10 mmHg have higher SBP, higher prevalence of hypertension, larger male sex ratio, higher body mass index, higher pulse rate, and lower ABI (7). Simultaneous measurement of IASBPD ≥15 mmHg predicts an increase in all-cause mortality, while IASBPD≥15 mmHg or ≥10 mmHg predicts an increase in cardiovascular mortality (8). Recent analysis of data from the Inter-arm Blood Pressure Difference Individual Participant Data (INTERPRESS-IPD) Collaboration reported aggregated longitudinal hard outcomes from over 50,000 participants, in which the IASBPD was associated with cardiovascular risk, even after adjusting for commonly used cardiovascular risk scores (1). IASBPD is also a predictor of coronary artery disease (9). The prevalence of IASBPD in participants with left ventricular hypertrophy and left ventricular remodeling was overall three times higher than in their normal counterparts (10). Furthermore, some studies have shown positive associations between IASBPD and chronic kidney disease (11), as well as cerebrovascular disease (12). Therefore, identifying risk factors for IASBPD is crucial for controlling the incidence of cardiovascular diseases.

Although some studies suggest that the male-to-female ratio is higher among populations with an IAD≥10 mmHg (7), there is currently limited research on the influencing factors of IAD across different gender groups.In humans, regardless of race or ethnicity, men generally have higher blood pressure than women. Moreover, this difference has also been observed in various species, including dogs, rats, mice, and chickens (13). The current research findings indicate that a connection between the existence of differences in IAD, handedness, gender, and phase of the ovarian cycle, which may suggest an asymmetrical ambulatory influence of sex hormones on blood pressure regulation (14). Although some studies have hinted that obesity-related indicators might be a significant risk factor for IAD (3), the majority of these studies have failed to account for gender-based differences. This study further explores the risk factors for inter-arm systolic blood pressure difference among different genders, aiming to assist individuals of different genders in identifying risk factors in a timely and effective manner.

Assessment parameters for obesity include BMI, waist circumference (WC), NC, and VFA, all of which are quantifiable via bio-electrical impedance analysis (BIA). This method is characterized by high accuracy, safety and non-invasiveness, and rapid measurement. Previous studies have demonstrated a strong association between visceral adipose tissue and cardiovascular risk factors (15). Studies have found that age, systolic blood pressure, triglycerides and WC are important predictors of CF-PWV, while NC and gender are significant predictors of Carotid-radial pulse wave velocity (PWVcr) (16). These findings highlight the need to measure not only waist circumference but also neck circumference to better stratify individuals and identify those at increased cardiometabolic risk, as upper-body subcutaneous fat represents a novel and easily measurable site of fat deposition.

## Materials and methods

### Object of study

A retrospective analysis was conducted on the data of individuals who underwent body composition testing in our department from 2019 to 2022, and also completed measurements of peripheral arterial blood pressure, biochemical tests, and other relevant examinations at our hospital's health check-up center or outpatient clinic. Individuals meeting the following criteria were selected as the study subjects: ①no cardiovascular and cerebrovascular diseases or other major illnesses such as tumors, liver and kidney dysfunction, hematological diseases, thyroid diseases, or pregnancy;②able to work and live normally; ③no history of diabetes and fasting blood glucose levels not exceeding 7.0 mmol/L. A total of 931 individuals were included, including 508 males and 423 females, with ages ranging from 20 to 82 years and a mean age of (50.03 ± 9.97) years. This study obtained ethical approval (Approval No. 2022-NKZ-022) from the Clinical Technology Application Ethics Committee of the First Affiliated Hospital of Anhui Medical University.

### Anthropometry

Use unified tools and methods to measure the height and weight of the subjects. Height should be measured to the nearest 0.1 centimeter, and weight to the nearest 0.1 kilogram. The VS-1000 arteriosclerosis detector from Fukuda Corporation of Japan is used to measure blood pressure in the limbs and the ABI. In a quiet environment, subjects are positioned supine with their limbs naturally relaxed and extended. Blood pressure cuffs are placed on the upper arms and ankles, and electrocardiogram electrodes are placed on both wrists. Simultaneously, a heart sound sensor is placed at the second intercostal space in the precordial region, with the criterion being a stable heart sound baseline and clear distinction between the first and second heart sounds. After completing the preparations, input the basic information of the subjects, start the measurement, and automatically calculate the ABI test results. IASBPD is defined as the absolute value of the difference between the synchronously measured systolic blood pressure (SBP) in the right upper limb and the left upper limb. Hypertension is defined according to the standards outlined in the Chinese Guidelines for the Prevention and Treatment of Hypertension, which includes self-reported history of hypertension, SBP≥140 mmHg, diastolic blood pressure (DBP) ≥ 90 mmHg, or current use of medication for hypertension.

### Biochemical project testing

Collect fasting venous blood (after fasting for 8 h or more) from the participants, place it in a tube that promotes coagulation, and utilize the RL7600 (Hitachi, Japan) fully automatic biochemical analyzer to measure low-density lipoprotein cholesterol (LDL-C), high-density lipoprotein cholesterol (HDL-C), triacylglycerol (TG), total cholesterol (TC), fasting blood glucose, and uric acid levels. Blood routine samples are analyzed using the LH750 automatic hematology analyzer (Beckman Coulter, USA). According to the Guidelines on Prevention and Treatment of Blood Lipid Abnormality in Chinese Adults (2007), the diagnostic criteria for dyslipidemia are defined as follows: LDL-C > 3.37 mmol/L, HDL-C < 1.04 mmol/L, TC > 5.18 mmol/L, or TG > 1.70 mmol/L. Abnormal levels of any or all of these lipids in the plasma are classified as dyslipidemia.

### Determination of human body composition

Using the Iobody770 body composition analyzer (Bio space, South Korea) bioelectrical impedance method to measure body composition such as body mass index (BMI), waist circumference (WC), neck circumference (NC), abdominal circumference (AC), and visceral fat area (VFA). Testers have received standardized professional training, and the testing is conducted in the morning. For the measurement, the subject should be in a fasting state or with an empty bladder and bowels, wear light clothing, and expose the electrode contact areas on the limbs. The measurement shall be conducted after 5 min of rest to ensure the accuracy of the results.

### Statistical analysis

Statistical analysis was performed using SPSS 25.0 software, and Quartile Scatter Plot were generated with GraphPad Prism 8.0. Normally distributed measurement data were expressed as mean ± standard deviation (x¯ ± s), and two-independent-sample *t*-tests were applied for between-group comparisons. Categorical data were presented as percentages (%), with *χ*^2^ tests used for intergroup comparisons. Multivariable analysis was conducted via multiple linear regression analysis. Statistically significant indicators were further analyzed using univariate and multivariate logistic regression (stepwise regression method). The diagnostic efficacy of independent influencing factors was evaluated by receiver operating characteristic (ROC) curve analysis. *P* < 0.05 was considered statistically significant.

## Results

### Baseline characteristics of the study population

There were 758 (81.4%) participants in the group with IASBPD≤10 mmHg and 173 (18.6%) in the group with IASBPD≥10 mmHg. Participants with IASBPD≥10 mmHg had higher obesity-related indices, including MI, WC, NC, AC, and VFA (*P* < 0.05). Compared to the group with IASBPD < 10 mmHg, the group with IASBPD≥10 mmHg had higher levels of GLU, UA, WBC, monocytes, and lymphocytes (*P* < 0.05), as well as lower ABI values on both sides (*P* < 0.05). Additionally, participants with IASBPD≥10 mmHg had a higher prevalence of hypertension and hyperlipidemia (*P* < 0.05). See Table 1.

**Table 1:** Baseline characteristic.

Characteristic	<10 mmHg(*n* = 758)	≥10 mmHg(*n* = 173)	*t*/*χ*^2^	*P*
Age (year)	49.91 ± 9.87	50.55 ± 10.40	0.762	0.446
Men, *n* (%)	396 (52.2)	112 (64.7)	8.873	0.003
BMI (kg/m^2^)	24.14 ± 3.42	25.34 ± 3.69	4.094	<0.001
WC (cm)	85.66 ± 9.53	88.72 ± 10.57	3.736	<0.001
NC (cm)	35.94 ± 2.87	37.02 ± 3.07	4.424	<0.001
AC (cm)	30.72 ± 3.03	31.86 ± 3.29	4.388	<0.001
VFA(cm^2^)	84.50 ± 32.42	91.61 ± 37.49	2.523	0.012
BF%	27.55 ± 6.58	27.87 ± 7.33	0.526	0.599
SBP-Left (mmHg)	127.17 ± 15.21	133.40 ± 17.55	4.312	<0.001
SBP-Right (mmHg)	127.35 ± 15.36	135.75 ± 18.16	6.257	<0.001
ABI-left (%)	110.89 ± 7.61	105.83 ± 8.08	−7.805	<0.001
ABI-right (%)	111.94 ± 7.74	108.38 ± 7.92	−5.423	<0.001
GLU (mmol/L)	5.60 ± 0.52	5.71 ± 0.51	2.339	0.020
UA (*μ*mol/L)	327.64 ± 87.73	347.23 ± 89.77	2.638	0.008
TC (mmol/L)	4.92 ± 0.92	4.97 ± 0.92	0.689	0.491
TG (mmol/L)	1.54 ± 0.92	1.81 ± 1.10	2.988	0.003
LDL-C (mmol/L)	2.93 ± 0.85	3.00 ± 0.86	0.939	0.348
HDL-C (mmol/L)	1.41 ± 0.38	1.30 ± 0.33	−3.957	<0.001
WBC(10^9/L)	6.03 ± 1.60	6.38 ± 1.90	2.259	0.025
Monocyte(10^9/L)	0.35 ± 0.12	0.37 ± 0.12	2.697	0.007
Lymphocyte(10^9/L)	2.00 ± 0.56	2.13 ± 0.69	2.291	0.023
Neutrophil(10^9/L)	3.51 ± 1.26	3.69 ± 1.44	1.635	0.102
Hypertension, *n* (%)	258 (34.0)	105 (60.7)	42.075	<0.001
Dyslipidemia, *n* (%)	240 (31.7)	66 (38.2)	2.687	0.101

BMI, body mass index; WC, waist circumference; NC, neck circumference; AC, arm circumference; VFA, visceral fat area; BF%, body fat percentage; ABI, ankle-brachial index; SBP, systolic blood pressure; GLU, glucose; UA, uric acid; TC, total cholesterol; TG, triacylglycerol; LDL-C, low-density lipoprotein cholesterol; HDL-C, high-density lipoprotein cholesterol; WBC, white blood cell.

### Factors influencing IASBPD

We conducted a multivariate linear stepwise regression analysis with age, BMI, GLU, UA, ABI-left (%), ABI-right (%), TG, HDL, WBC, monocytes, lymphocytes, NC, WC, AC, and VFA as independent variables, and the difference between IASBPD as the dependent variable. The results indicated that IASBPD was positively correlated with NC, age, and lymphocytes, but negatively correlated with the ABI-left (%). See Table 2.

**Table 2:** Multiple linear regression analysis of indicators related to IASBPD.

Parameter	*β*'	SE	t	*P*
ABI-left(%)	−0.335	0.019	−10.760	0.000
NC	0.182	0.052	5.769	0.000
Age	0.085	0.015	2.741	0.006
Lymphocyte	0.070	0.257	2.243	0.025

IASBPD, inter-arm systolic blood pressure difference; ABI, ankle-brachial index; NC, neck circumference.

A binary logistic regression analysis was conducted with the ABI-left%, NC, age, lymphocytes, sex(man=1,woman=0),Hypertension(N-Hypertension=0,Hypertension=1), as independent variables, and the presence of abnormal inferior abdominal aortic IASBPD(IASBPD < 10 = 0, IASBPD≥10 = 1) as the dependent variable. The results showed that in the general population, an increased ABI was a protective factor for IASBPD, while increased NC and hypertension were risk factors for IASBPD. However, an increase in NC is a risk factor for an elevated IASBPD in males, while advanced age is a risk factor for increased IASBPD in females. See Table 3.

**Table 3:** Multivariate logistic regression analysis of indicators related to IASBPD.

Parameter	Sample size	Model	Variable	B	SE	*χ*2	P	OR(95%CI)
Total people	931	1	ABI-left(%)	−0.087	0.012	53.603	<0.001	0.917（0.896−0.939）
		2	ABI-left(%)	−0.097	0.012	61.111	<0.001	0.907（0.886–0.930）
		1	NC	0.126	0.029	18.695	<0.001	1.135（1.071–1.201）
		2	NC	0.126	0.032	15.255	<0.001	1.134（1.065–1.208）
		1	Hypertension	1.096	0.174	39.907	<0.001	2.992（2.130–4.205）
		2	Hypertension	1.007	0.188	28.783	<0.001	2.739（1.895–3.957）
Male	508	1	ABI-left(%)	−0.121	0.017	52.7356	<0.001	0.886（0.858–0.915）
		2	ABI-lef(%)	−0.128	0.017	54.569	<0.001	0.880（0.850–0.910）
		1	NC	0.161	0.048	11.045	0.001	1.174（1.068–1.291）
		2	NC	0.192	0.054	12.805	<0.001	1.211（1.090–1.345）
		1	Hypertension	0.824	0.219	14.168	<0.001	2.280（1.484–3.501）
		2	Hypertension	0.666	0.242	7.591	0.006	1.947（1.212–3.128）
Female	423	1	Hypertension	1.424	0.288	24.519	<0.001	4.153（2.364–7.297）
		2	Hypertension	1.366	0.305	20.043	<0.001	3.921（2.156–7.132）
		1	ABI-left(%)	−0.057	0.019	9.473	0.002	0.944（0.910–0.979）
		2	ABI-lef(%)	−0.073	0.020	13.273	<0.001	0.930（0.894–0.967）
		1	Age	0.038	0.014	6.983	0.008	1.039（1.010–1.068）
		2	Age	0.030	0.015	3.816	0.051	1.030（1.000–1.062）

Model 1 is a single factor analysis; Model 2 was calibrated based on Model 1, adjusting for ABI-left(%), ABI-right(%), NC, Age, Lymphocyte, BMI, gender(female=0,male=1), Hypertension(N-Hypertension=0,Hypertension=1), uric acid, blood glucose, Total cholesterol, Triacylglycerol, Low-density lipoprotein cholesterol, High-density lipoprotein cholesterol. IASBPD: inter-arm systolic blood pressure difference; ABI: Ankle-Brachial Index; NC: Neck Circumference; BMI: Body Mass Index.

### Factors influencing IASBPD in different gender populations

**Figure 1:**
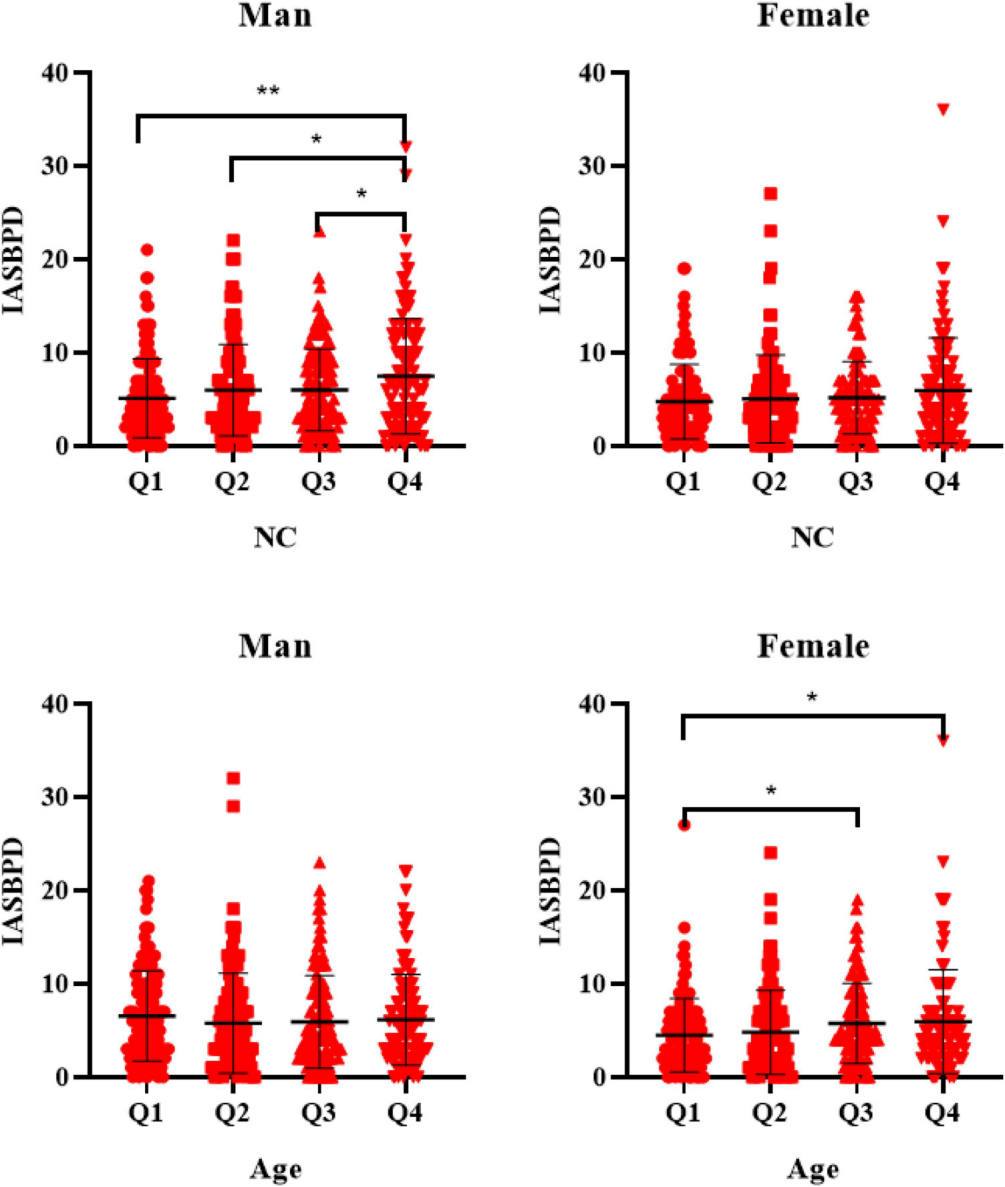
IASBPD values grouped by quartiles of NC and Age. IASBPD, inter-arm systolic blood pressure difference; NC, neck circumference.

In the quartile grouping based on NC, IASBPD value in the Q4 group was significantly higher than that in the other groups among males; whereas in the quartile grouping based on age,the IASBPD values in the Q4 and Q3 groups were significantly higher than those in the Q1 group among females. See Figure 1.

**Figure 2:**
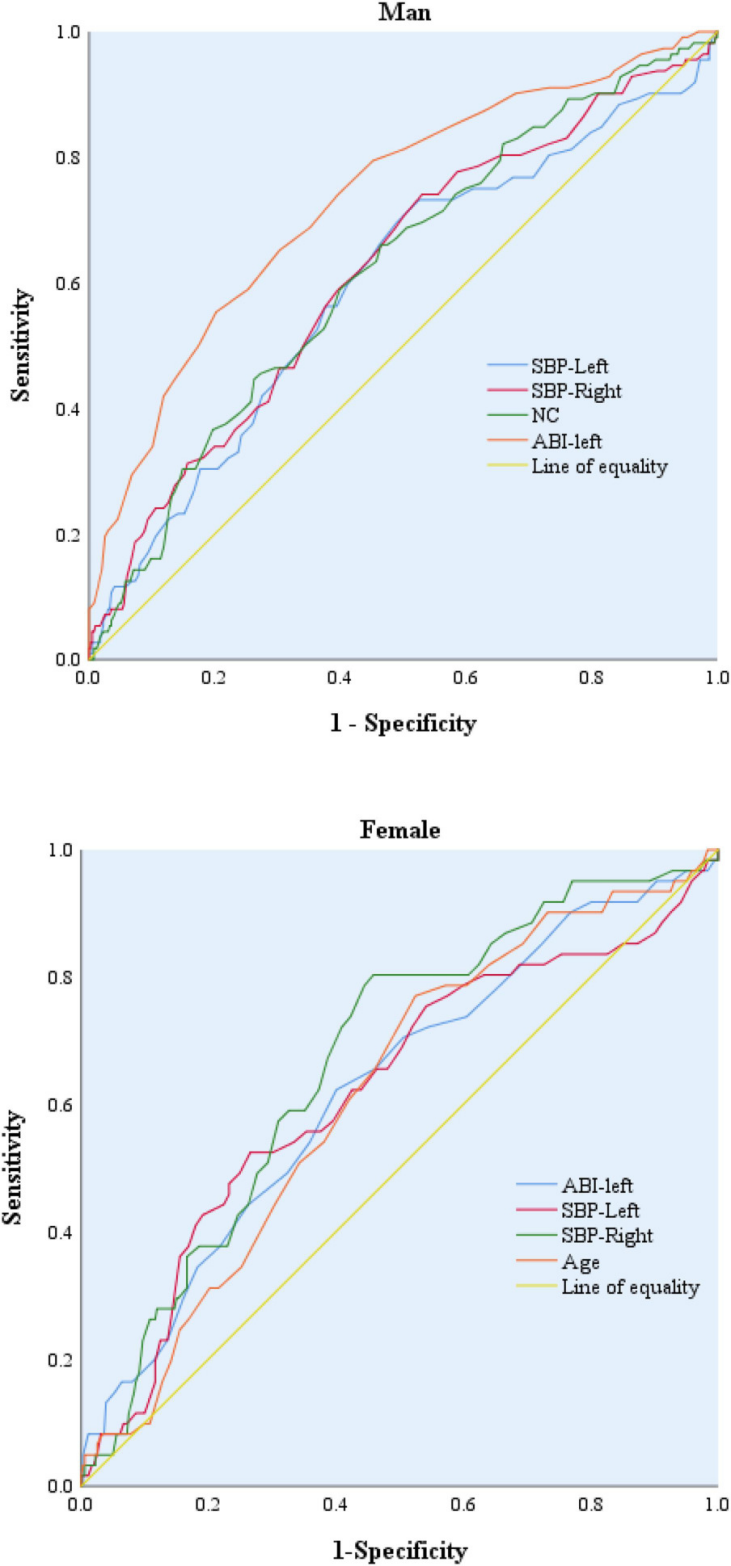
Forest plot of risk factors for IASBPD. IASBPD, inter-arm systolic blood pressure difference; ABI, ankle-brachial index; NC, neck circumference.

The ROC curve indicates that ABI-left, right upper arm systolic blood pressure, and left upper arm systolic blood pressure all exhibit certain predictive performance for the increase in IASBPD values in both male and female populations.However, in the male population, NC(AUC: 0.620, 95%CI: 0.561–0.678, *P* < 0.001) exhibits certain predictive performance for the increase in IASBPD, the optimal cut-off value of NC is 37.75 (sensitivity: 66.1%, specificity: 53.5%). In the female population, Age (AUC: 0.618, 95%CI: 0.545–0.690, *P* = 0.003) shows certain predictive performance for the elevation of IASBPD, the optimal cut-off value of Age is 49.5 (sensitivity: 77.0%, specificity: 47.5%). See Figure 2.

## Discussion

The findings of this study revealed that an IASBPD is a common phenomenon, with 17.5% of the observed subjects exhibiting an IASBPD of ≥10 mmHg. A study conducted in a Chinese community population showed that the prevalence rate of IASBPD≥10 mmHg was 12.8% (17). Another study involving rural and urban community residents from China utilized the Vascular Profiler-1000 device to measure four-limb blood pressure values. The findings indicated that 15% of subjects exhibited an IAD ≥10 mmHg (7). A study from Japan reported that 9.1% of the observed subjects exhibited an IASBPD≥10 mmHg (18). A study from South Africa revealed that 14.78% of participants exhibited an IASBPD≥10 mmHg (19). Although the prevalence of IASBPD varies across regions, it should be assessed in all patients due to its association with the progression and prognosis of multiple diseases. Elevated IASBPD is a significant predisposing factor for various cardiovascular and other conditions. Detecting and managing modifiable risk factors for increased IASBPD may help reduce its prevalence and associated comorbidities (12).

The findings of this study also revealed that participants with an IASBPD≥10 mmHg exhibited significantly higher obesity-related indicators, including: BMI, WC, NC, AC and VFA (*P* < 0.05). In several studies, patients with significant IASBPD were more obese compare to other patients. Those with IAD≥10 mmHg had higher systolic blood pressure (SBP), higher prevalence of hypertension, larger male gender ratio, bigger body mass index, higher pulse rate and lower ABI (7). A positive correlation was observed between IAD and Mid-Upper Arm Circumference(MUAC) as well as NC, suggesting that these body circumference measurements may serve as indicators of elevated IAD (19). Despite similar BMI among participants, patients with an IASBPD > 10 mmHg exhibited significantly higher levels of VFA (4). NC is demonstrated that a strongest correlation with both inter-arm systolic difference (IAS) and IAD; MUAC showed a moderate association with IAS but exhibited a weaker association with IAD (3). Patients with obesity are more likely to have cardiovascular events, including atherosclerosis, CAD and stroke, which are significantly related to the increase of IABPD (3). Obesity is considered a risk factor of elevated IASBPD and should be treated for its adverse cardiovascular outcome. The associated factors with the presence of IAD have been controversial in previous studies duo to diversities in studied population, demographic characteristics, ethnicity, and IAD measurement method.

This study excluded individuals with abnormal levels of blood glucose or a history of diabetes. Results showed that participants with IASBPD≥10 mmHg exhibited: higher prevalence of hypertension, higher prevalence of dyslipidemia, elevated inflammatory markers. It has been reported that IASBPD are observed in individuals with hypertension (20). The probability of IASBPD ≥10 mmHg significantly increases with advancing age and higher severity of hypertension (21). The prevalence of IAD ≥ 10 mmHg is significantly higher in hypertensive patients compared to the general population (22). Individuals with IADSBP ≥ 10 mmHg exhibit higher BMI, elevated triglyceride levels and lower high-density lipoprotein (HDL) cholesterol levels (5). Chronic inflammation can trigger various diseases, including NAFLD, CVD, and obesity, which are leading causes of global disability and mortality (23). Obesity is closely linked to inflammation, and both are critical factors in the pathogenesis and progression of CVD (24). Neoantigens, the NLRP3 inflammasome, cytokines, and others can promote immune activation in hypertensive patients (25). Current evidence has confirmed that IASBPD is associated with conditions such as obesity, hypertension, hyperlipidemia, and others, its relationship with inflammation remains understudied, and further research is warranted to explore this linkage.

Further analysis indicates that IASBPD is positively associated with NC, age, and lymphocytes, but negatively associated with ABI. Elevated ABI acts as a protective factor for IASBPD in populations of varying ages and genders. Other studies have also found that abnormal IASBPD is associated with markers of cardiovascular disease, such as the ABI (26). Hypertensive patients with higher IASBPD had lower ABI (27). Consistent with the findings of this study, other research has indicated that patients with IASBPD≥10 mmHg tend to exhibit a lower ABI (7). A low ABI value reflects poor blood flow status in the lower extremity arteries, which may be interrelated with arterial system abnormalities indicated by an increased IASBPD. Therefore, in clinical practice, simultaneous measurement of both IASBPD and ABI can provide physicians with more comprehensive cardiovascular health assessment information.

This study further investigated the risk factors for IASBPD among populations of different genders. The results showed that an increase in NC and age were risk factors for the elevation of IASBPD in the male and female populations, respectively. ROC curve analysis also confirmed that NC and age exhibited certain predictive capabilities for the increase in IASBPD values in the male and female populations, respectively. NC, as a measure of body fat, is used to assess upper-body subcutaneous fat. It is more stable and simpler to measure than waist circumference, unaffected by food intake, body posture, and respiration. It overcomes the limitations of the aforementioned traditional anthropometric indicators and is also considered an anthropometric measure for abdominal obesity (28). High NC was associated with an increased risk of early stage atherosclerosis in Chinese adults (29). The Framingham Heart Study found that NC was positively associated with cardiometabolic risk, and this correlation is independent of BMI and visceral fat, this suggests that the upper-body subcutaneous fat may add additional risk beyond general and visceral fat (30). PWVcr is one of the crucial indicators for evaluating arterial stiffness, with NC and gender serving as significant predictors of PWVcr (16). Even after adjusting for covariates such as BMI, WC, or waist-to-hip ratio, NC in adults from Northeast China still exhibits a significant correlation with arterial blood pressure and hypertension risk (31). Therefore, NC may play an independent role in predicting hypertension and IASBPD beyond the classical anthropometric indices.

There are certain differences in the influencing factors of IASBPD among populations of different genders, and the reasons underlying these gender differences require further investigation. Although studies have shown that differences in IASBPD are common among healthy young subjects, they did not account for factors such as gender, handedness, or the stage of the ovarian cycle (32). Other studies have indicated that there is no significant increase in systolic blood pressure among dominant-hand (DOM) populations, thereby clarifying that handedness, as a factor, generally does not need to be considered in clinical assessments (33). Research findings indicate that there are functional interactions among handedness, blood pressure, gender, and the stage of the ovarian cycle, which may involve asymmetric regulation of blood pressure control by sex hormones (14). In females, renin-angiotensin-aldosterone system (RAAS) is influenced significantly by oestrogen status, RAAS components such as plasma renin, fluctuate throughout the menstrual cycle in response to altering levels of estradiol (34). Ang II-mediated pressor responses are ameliorated via angiotensin type 2 receptor (AT2R) activation in female but not male murine models (35). Moreover, this inhibitory effect tends to diminish with advancing age and reproductive senescence, and can be restored following estrogen replacement therapy (36). Therefore, advancing age and estrogen exposure are crucial mediating factors in the blood pressure-lowering effects regulated by the RAAS pathway, and they may hold particular significance in the development of hypertension among post-menopausal women. This study suggests that advanced age is a risk factor for elevated IASBPD in women. However, a limitation of this study is that it did not collect data on the menopausal age of female participants or the fluctuations in blood pressure before and after menopause.

Currently, we cannot find any other research reports on the influencing factors of IASBPD among different gender groups, and our research conclusions need to be verified and explored with more population data, such as data from populations of different countries, ethnicities, and diseases.

## Data Availability

The raw data supporting the conclusions of this article will be made available by the authors, without undue reservation.
